# Analytical Agreement and Diagnostic Performance of a Commercial Point-of-Care Lipid Testing System for Lipid Profile and Atherogenic Ratios

**DOI:** 10.3390/diagnostics16193116

**Published:** 2026-09-25

**Authors:** Susana Guadalupe Guzmán-Aquino, Roberto Rivera-Sánchez, Karla Aidee Aguayo-Cerón, Manuel Solis-Navarro, Claudia Camelia Calzada-Mendoza, Rodrigo Romero-Nava, Rodolfo Pinto-Almazán, Adriana Flores-Miranda, Paola Berenice Zárate-Segura, Cruz Vargas-De-León

**Affiliations:** 1 Laboratorio de Modelación Bioestadística, Sección de Estudios de Posgrado e Investigación, Escuela Superior de Medicina, Instituto Politécnico Nacional, Plan de San Luis y Díaz Mirón s/n, Mexico City 11340, Mexico; lu.aquino128@gmail.com (S.G.G.-A.); manuelsolisup@gmail.com (M.S.-N.); 2 División de Investigación, Hospital Juárez de México, Av. Instituto Politécnico Nacional, Mexico City 07760, Mexico; cerdea321@yahoo.com.mx (R.R.-S.); afloresm.124@gmail.com (A.F.-M.); 3 Laboratorio de Investigación en Genética de Enfermedades Metabólicas, Escuela Superior de Medicina, Instituto Politécnico Nacional, Plan de San Luis y Díaz Mirón, Mexico City 11340, Mexico; aidee.aguayo@gmail.com (K.A.A.-C.); rromeron@ipn.mx (R.R.-N.); 4 Laboratorio de Señalización Intracelular, Sección de Estudios de Posgrado e Investigación, Escuela Superior de Medicina, Instituto Politécnico Nacional, Plan de San Luis y Díaz Mirón, Mexico City 11340, Mexico; cccalzadam@yahoo.com.mx; 5 Sección de Estudios de Posgrado e Investigación, Escuela Superior de Medicina, Instituto Politécnico Nacional, Plan de San Luis y Díaz Mirón, Mexico City 11340, Mexico; rodolfopintoalmazan@gmail.com; 6 Laboratorio de Medicina Traslacional, Sección de Estudios de Posgrado e Investigación, Escuela Superior de Medicina, Instituto Politécnico Nacional, Plan de San Luis y Díaz Mirón s/n, Mexico City 11340, Mexico

**Keywords:** agreement analysis, dyslipidemia, lipid profile, atherogenic ratios, point-of-care lipid testing device

## Abstract

**Background:** Point-of-care testing (POCT) devices offer a rapid alternative for lipid assessment, but comprehensive analytical validation against reference laboratory methods remains limited. **Objective:** This study aimed to evaluate the agreement, systematic bias, and diagnostic performance of the Mission^®^ Cholesterol 3-in-1 system for lipid profile measurements and atherogenic ratios. Methods: A prospective cross-sectional agreement study was conducted among 120 participants. Lipid profiles were measured using eleven Mission^®^ Cholesterol 3-in-1 analyzers and compared with those measured using the reference laboratory method. Agreement was assessed using the intraclass correlation coefficient (ICC), concordance correlation coefficient (CCC), coefficient of determination ($R^{2}$), root mean square error (RMSE), mean absolute error (MAE), percentage bias, Bland–Altman plots, Passing–Bablok regression, and diagnostic accuracy. **Results:** Triglycerides demonstrated excellent agreement (ICC = CCC = 0.972; bias = 5.19%), whereas total cholesterol, HDL-C, non-HDL-C, and LDL-C showed good agreement (ICC range: 0.817–0.854). Among the atherogenic ratios, TG/HDL-C (ICC = 0.932) and TG/LDL-C (ICC = 0.931) exhibited the highest concordance. Passing–Bablok regression revealed constant bias for all analytes, except for LDL-C estimated using the Sampson equation and the TG/LDL-C ratio, while proportional bias was observed for all analytes, except for triglycerides and the TG/LDL-C ratio. Despite these systematic differences, diagnostic performance remained excellent, with sensitivities of 94.29–98.33% and specificities of 68.33–91.80%. **Conclusion:** The Mission^®^ Cholesterol 3-in-1 system shows good analytical agreement with laboratory testing, particularly for triglycerides and the HDL-C, TG/HDL-C, and TG/LDL-C ratios. Although systematic bias limits complete interchangeability with reference methods, its excellent diagnostic performance supports its use as a rapid point-of-care screening tool, especially in resource-limited settings.

## 1. Introduction

Dyslipidemia or hyperlipidemia is a blood lipid disorder that can manifest as excessive elevation of total cholesterol (hypercholesterolemia), high concentrations of low-density lipoprotein cholesterol (LDL-C), low concentrations of high-density lipoprotein cholesterol (HDL-C), or elevated triglycerides (hypertriglyceridemia). These alterations contribute to the development and progression of atherosclerotic cardiovascular disease and represent important modifiable factors in cardiovascular prevention [1,2].

Elevated triglycerides (TGs) and reduced HDL-C are two of the five dyslipidemias commonly considered in the identification of metabolic syndrome in adults and adolescents. Their coexistence reflects an atherogenic and insulin-resistant metabolic profile associated with an increased risk of type 2 diabetes and cardiovascular disease (CVD) [3,4]. Accurate assessment of these lipid abnormalities is therefore relevant not only for conventional dyslipidemia screening but also for the broader evaluation of cardiometabolic risk.

Dyslipidemia is a common public health problem worldwide, although its prevalence varies according to the lipid abnormality evaluated, diagnostic thresholds, and study population characteristics. A recent systematic review and meta-analysis of more than 200 population-based studies estimated global adult prevalences of 38.4% for reduced HDL-C, 28.8% for hypertriglyceridemia, 24.1% for hypercholesterolemia, and 18.9% for elevated LDL-C [5]. These abnormalities contribute substantially to atherosclerotic cardiovascular disease, which remains the leading cause of mortality and disability in developed societies. In 2022, cardiovascular diseases accounted for an estimated 19.8 million deaths, representing approximately 32% of all global deaths [6].

In Mexico, heart disease decreased by 7.8 deaths per 100,000 population in 2025 compared with the same period in 2024 but remained the leading cause of death, both overall and by sex [7]. Dyslipidemia is also highly prevalent among Mexican adults. Analysis of ENSANUT 2012 showed that reduced HDL-C and elevated LDL-C were the most frequent lipid abnormalities, while approximately one in four adults had hypercholesterolemia without a previous diagnosis [8]. More recently, ENSANUT 2022 reported that 30.6% of adults had a previous diagnosis of hypercholesterolemia [9]. Together, these findings suggest that a substantial proportion of lipid abnormalities remain undetected, reinforcing the need for accessible and reliable screening strategies.

According to American Heart Association recommendations, maintaining healthy cholesterol levels is important for reducing the risk of heart disease and stroke. It is recommended that adults older than 20 years check their lipid profile every 4 to 6 years, with the interval adjusted according to age, previous results, comorbidities, and overall cardiovascular risk. A standard lipid profile includes total cholesterol (TC), TGs, HDL-C, LDL-C, and cholesterol calculated from non-HDL lipoproteins (non-HDL-C) [10].

In addition to individual lipid concentrations, lipid ratios may provide complementary information by integrating the relationships among triglyceride-rich lipoproteins, atherogenic cholesterol fractions, and HDL-C. The TG/HDL-C ratio has been extensively investigated as an indirect marker of insulin resistance and an atherogenic lipid phenotype, and it has been associated with metabolic syndrome and cardiovascular risk [11,12]. Nevertheless, its magnitude and optimal cut-off values may vary according to sex, age, ethnicity, and metabolic characteristics, and it should therefore be interpreted within the context of the population evaluated.

The TC/HDL-C and LDL-C/HDL-C ratios express the balance between the cholesterol contained in potentially atherogenic lipoproteins and the cholesterol transported by HDL particles. Higher values have been associated with an unfavorable lipid profile, atherosclerotic burden, and an increased cardiovascular risk [13,14]. Similarly, the non-HDL-C/HDL-C ratio compares cholesterol contained in all apoB-containing lipoproteins with HDL-C and may provide a broader representation of the balance between atherogenic and protective lipid fractions. TG/LDL-C has been less investigated but has been proposed for identifying a higher proportion of small-density LDL particles in patients with type 2 diabetes and hypertriglyceridemia [15]. However, its diagnostic thresholds and clinical relevance have not been established in different populations, and additional validation is required. Its calculation from point-of-care measurements could also extend the potential usefulness of portable lipid analyzers beyond the identification of isolated lipid abnormalities. Nevertheless, these derived indices should complement rather than replace the interpretation of individual lipid concentrations and comprehensive cardiovascular risk assessment.

Accurate and reliable measurement of the lipid profile is essential for assessing cardiovascular risk and monitoring patient treatment. Traditionally, these analyses are performed in clinical laboratories using high-precision and sophisticated equipment; however, access to centralized laboratory testing may be limited by infrastructure, cost, transportation, or the need for patients to return for their results. Because of these drawbacks, point-of-care testing (POCT) devices have emerged as an alternative, offering potential advantages including portability; rapid result availability; small sample volume requirements; and the possibility of performing measurements in primary care, community, epidemiological, and resource-limited settings [16,17]. POCT lipid systems commonly use disposable test strips to quantify lipid components from whole-blood samples. Depending on the system, TC, TG, and HDL-C may be measured directly, while LDL-C or some ratios may be calculated from the primary measurements.

Despite their operational advantages, the analytical performance of POCT lipid systems is not uniform across devices or analytes. Previous validation studies have reported differences in agreement, imprecision, systematic bias, and diagnostic accuracy when portable systems are compared with standardized laboratory methods [18,19,20]. Nevertheless, most available validations have included relatively small study populations (49–63 participants), evaluated only a single analyzer, and relied on a limited set of agreement statistics. As a result, evidence regarding the analytical reproducibility and overall clinical performance of these devices remains limited. A more comprehensive validation framework incorporating multiple analyzers and complementary analytical and diagnostic performance metrics is therefore warranted.

The purpose of this study is to evaluate the agreement of a lipid testing analyzer (Mission^®^ Cholesterol 3-in-1) in a clinical setting. Through this comparison, we seek to determine whether POCT can be considered a reliable alternative for lipid profile evaluation in scenarios where access to laboratory equipment is limited or impractical.

## 2. Materials and Methods

### 2.1. Study Design

A cross-sectional concordance study was conducted. Analysis and reporting of the agreement between methods were carried out following the recommendations of the Guidelines for Reporting Reliability and Agreement Studies (GRRAS) [21].

### 2.2. Participants

Consecutive sampling was used to recruit adults aged 18 years or older who were undergoing routine lipid profile testing (TG, TC, HDL-C, and LDL-C). Individuals who had not completed an 8–10 h fasting period or who were taking vitamin C supplements were excluded from the study.

### 2.3. Blood Sample

Venous blood samples were collected from each participant. The laboratory reference measurements were performed using venous blood samples with a standardized laboratory analyzer. For the POCT assessment, an aliquot of the same venous whole-blood sample was used for lipid measurements with the Mission^®^ Cholesterol 3-in-1 system. Laboratory-based and point-of-care measurements were performed by different operators, ensuring independent assessment of the samples.

### 2.4. Laboratory

Lipid profile measurements were performed using an iLab Taurus Plus analyzer (Werfen Instrumentation Laboratory, Bedford, MA, USA) [22].

The analyzer was subjected to routine quality control using Eightcheck-3WP X-TRA standardized commercial sera (Sysmex America, Inc., Lincolnshire, IL, USA), at low, normal, and high concentration levels, together with Bio-Rad Controls 1 and 2 (Bio-Rad Laboratories, Irvine, CA, USA).

Lipid profile measurements were performed under controlled environmental conditions, with a mean relative humidity of 48.8% (range, 35–55%) and a mean ambient temperature of 23.7 °C (range, 20–25 °C). All measurements were obtained at a constant altitude of 2240 m above sea level (Mexico City), in accordance with the manufacturers’ recommended operating specifications for the laboratory analyzer and the POCT system.

### 2.5. Mission^*®*^ Cholesterol 3-in-1

In this study, a total of 11 Mission^®^ Cholesterol 3-in-1 devices were used. The Mission^®^ Cholesterol 3-in-1 system is a portable in vitro diagnostic system designed by the manufacturer to measure lipid parameters using different specimen types, including capillary blood, whole blood, plasma, or serum. In the present study, however, the POCT measurements were performed exclusively using venous whole-blood samples. The system measures total cholesterol (TC), HDL-C, and triglycerides (TGs) simultaneously.

The POCT device is based on electrochemistry and reflectometry technology. Measurement is carried out using a timed endpoint. In this process, the device detects and monitors changes in light absorbance at a wavelength of 635 nm over a fixed time interval. The variation in absorbance is proportional to the concentration of lipids present in the sample.

The device is designed to operate within a temperature range of 15 to 40 °C, ensuring accuracy and stability of the measurements under various environmental conditions [23].

### 2.6. Estimation of LDL Cholesterol

For the Mission^®^ device, we used the Friedewald formula [24]:

$$\mathrm{LDL}-C=\mathrm{TC}-\mathrm{HDL}-C-\frac{\mathrm{TG}}{5.0}$$

which estimates LDL-C based on measured values of TC, TG, and HDL-C. We also used the Sampson formula [25]:

$$\mathrm{LDL}-C=\frac{\mathrm{CT}}{0.948}-\frac{\mathrm{HDL}-C}{0.971}-\left[\frac{\mathrm{TG}}{8.56}+\frac{\mathrm{TG}\times \mathrm{non}-\mathrm{HDL}-C}{2140}-\frac{{\mathrm{TG}}^{2}}{16,100}\right]-9.44$$

and the Martin formula [26]:

$$\mathrm{LDL}-C=\mathrm{TC}-\mathrm{HDL}-C-\frac{\mathrm{TG}}{\mathrm{AF}}$$

where AF is an adjustable factor defined according to triglyceride levels (TG) and non-HDL-C levels, used to estimate LDL-C based on the parameters obtained from the Mission^®^ device. For all three equations, TC, HDL-C, TG, and non-HDL-C corresponded to the values measured by the Mission^®^ device.

### 2.7. Agreement Analysis

Agreement analysis was performed using several key statistical coefficients to assess the relationship and similarity between the obtained measurements. The intraclass correlation coefficient (ICC) was calculated to assess the agreement between measurements obtained with the Mission^®^ analyzer and the laboratory reference method. ICC values were categorized as follows: <0.50 indicated poor reliability, 0.50–0.75 moderate reliability, 0.75–0.90 good reliability, and ≥0.90 excellent reliability [27]. Additionally, the concordance correlation coefficient (CCC) was used to measure the degree of agreement between two measurement methods, adjusting for both precision and accuracy. The CCC values were classified according to the strength of the agreement as follows: poor correlation (<0.50), fair correlation (0.50–0.70), good correlation (0.70–0.90), and excellent correlation (>0.90) [28,29]. The coefficient of determination ($R^{2}$) was also calculated to measure the proportion of variability in the data of one variable explained by another. Furthermore, the root mean square error (RMSE) and mean absolute error (MAE) were used as error measures. The RMSE provides a measure of the average magnitude of prediction errors, while the MAE quantifies the average absolute errors, providing an overview of the overall precision of the measurements. Percent bias (%) was calculated as the average relative difference between measurements obtained using the POCT device and those obtained using the reference laboratory method:

$$\mathrm{Percent}\;\mathrm{bias}\;(\%)=\frac{1}{n}\sum_{i=1}^{n}\left(\frac{{\mathrm{LDL}-C}_{\mathrm{POCT},i}-{\mathrm{LDL}-C}_{\mathrm{ref},i}}{{\mathrm{LDL}-C}_{\mathrm{ref},i}}\times 100\right).$$

Bias-corrected and accelerated (BCa) 95% bootstrap confidence intervals for the ICC, CCC, $R^{2}$, RMSE, MAE, and percent bias were estimated from 1000 bootstrap resamples to provide robust measures of parameter uncertainty.

Scatter plots were used to visualize the relationship between the Mission^®^ analyzer and the laboratory reference method, while Bland–Altman plots were constructed to evaluate agreement, systematic bias, and the limits of agreement between the two methods [30,31].

Passing–Bablok regression analysis was performed to assess agreement between the Mission^®^ analyzer and the laboratory reference method. The regression intercept and slope were estimated together with their 95% confidence intervals (CIs). Constant bias was considered present when the 95% CI of the intercept did not include zero, while proportional bias was considered present when the 95% CI of the slope did not include one. An intercept equal to zero and a slope equal to one indicate perfect agreement between the two analytical methods [32,33].

### 2.8. Between-Device Variability

Between-device variability was evaluated using fitting linear mixed-effects models in which the analyzer was specified as a random intercept, thereby accounting for heterogeneity across the 11 Mission^®^ Cholesterol 3-in-1 devices. Individual models were constructed for TC, TG, HDL-C, and LDL-C. Variance components attributable to differences between devices and to residual within-device variation were estimated using restricted maximum likelihood (REML). The device-level intraclass correlation coefficient (${\mathrm{ICC}}_{\mathrm{device}}$) was derived as the proportion of the total variance explained by between-device differences as follows:

(1) $${\mathrm{ICC}}_{\mathrm{device}}=\frac{\sigma_{\mathrm{between}-\mathrm{device}}^{2}}{\sigma_{\mathrm{between}-\mathrm{device}}^{2}+\sigma_{\mathrm{residual}}^{2}}$$

Additionally, the between-device coefficient of variation (CV%) was computed from the mean concentration obtained for each analyzer and was used as a descriptive measure of variability across devices.

### 2.9. Lipid Profile

Specific cut-off values were defined for the components of the lipid profile. TC was considered elevated when it exceeded 200 mg/dL. TG was considered elevated starting at 150 mg/dL. For HDL-C, levels below 40 mg/dL were considered low. LDL-C levels were evaluated as elevated when they were greater than or equal to 100 mg/dL. Additionally, non-HDL cholesterol was calculated by subtracting HDL from total cholesterol, and values greater than or equal to 130 mg/dL were considered elevated [34,35].

### 2.10. Lipid Ratio

Four lipid ratios or atherogenic indices were defined and calculated to assess cardiovascular risk. The triglyceride/HDL-C ratio (TG/HDL-C) was obtained by dividing the triglyceride concentration by HDL-C, and a value greater than 3 was considered a marker of elevated risk [36]. The total cholesterol/HDL ratio (TC/HDL-C) was calculated by dividing total cholesterol by HDL-C, using a previously proposed threshold of 4 for cardiovascular risk stratification [37]. The non-HDL-C/HDL-C ratio was defined as non-HDL-C divided by HDL-C, with risk values considered above 2.67 [38]. The LDL-C/HDL-C ratio was calculated by dividing LDL cholesterol by HDL, with a previously proposed threshold of 2.17 used to identify high cardiovascular risk [39]. Finally, the TG/LDL-C ratio was calculated by dividing triglycerides by LDL-C, and a 2.52 (1.1 molar ratio) threshold was applied to identify individuals with an increased burden of small, dense LDL particles [15].

### 2.11. Accuracy Measures

To assess the diagnostic accuracy of the measurements, participants were classified into two groups according to established clinical cut-off values for total cholesterol, triglycerides, HDL-C, LDL-C, non-HDL-C, and the atherogenic ratios. Sensitivity and specificity were calculated as measures of diagnostic accuracy, and their 95% confidence intervals were estimated using the exact Clopper–Pearson method. The area under the receiver operating characteristic curve (AUC) was also calculated for each test to quantify its overall discriminative performance.

### 2.12. Sample Size

Sample size was estimated for a Bland–Altman agreement study using the method proposed by Lu et al. [40]. A two-sided significance level of α=0.05, a statistical power of 90% (β=0.10), an expected mean difference (μ) of 5 mg/dL, a standard deviation of the paired differences (σ) of 10 mg/dL, and a maximum clinically acceptable difference (δ) of 30 mg/dL were assumed, representing the expected agreement scenario for the measurements of total cholesterol or triglycerides. The standardized parameters μ/σ=0.5 and δ/σ=3.0 were used to identify the corresponding sample size scenario in Table 1 of Lu et al. [40]. Based on these assumptions, the study required a minimum sample size of 110 participants.

### 2.13. Statistical Analysis

Statistical analyses were performed using Microsoft Excel and R software (version 4.4.1). The following R packages were used: irr (for the ICC) [41], DescTools (for the CCC) [42], metrics (for accuracy metrics such as the MAE and RMSE) [43], methcomp (for passing–Bablok regression) [44], and boot (for bootstrapping and BCa confidence intervals) [45]. Bland–Altman plots were generated using the ggplot2 environment [46].

#### Ethical Approval Declarations

The study protocol was reviewed and approved by the Ethics and Research Committee under approval number ESM-CEI-03/19-03-2026. The study was conducted according to the principles of the Declaration of Helsinki. Written informed consent was obtained from all participants prior to enrollment.

## 3. Results

### 3.1. Sample Description

A total of 120 participants underwent routine lipid profile testing, including 77 women (64.2%). Participants ranged in age from 18 to 86 years, with a mean age of 49.0 years (SD, 19.39) (Table 1).

Lipid abnormalities were common in the study population, as 49.2% of participants had hypertriglyceridemia, 33.3% had hypercholesterolemia, and 24.2% had low HDL-C levels. Elevated non-HDL-C and LDL-C concentrations were observed in 50.0% and 58.3% of participants, respectively. Type 2 diabetes mellitus was present in 35% of participants, while 5.8% had arterial hypertension.

The median (Q1, Q3) triglyceride concentration was 149.0 mg/dL (101.0, 187.0), while the mean total cholesterol concentration was 180.0 mg/dL (SD = 40.27), the mean HDL-C was 48.9 mg/dL (SD = 11.26), the mean non-HDL-C was 131.1 mg/dL (SD = 36.1), and the mean LDL-C was 108.8 mg/dL (SD = 34.6).

The median LDL-C/HDL-C ratio was slightly above the previously proposed threshold for coronary artery disease risk in population-based studies, while the median TG/HDL-C, TC/HDL-C, and TG/LDL-C ratios remained below their reference thresholds. Nevertheless, the upper quartiles of all four lipid ratios indicated that a substantial proportion of participants presented values compatible with an increased cardiometabolic risk.

### 3.2. Agreement Analysis

Scatter plots demonstrated a strong positive linear relationship between the lipid measurements obtained with the Mission^®^ device and those generated by the reference laboratory method for total cholesterol, triglycerides, HDL-C, and non-HDL-C (Figure 1). The Bland–Altman analysis also showed good agreement between both methods, with most observations falling within the 95% limits of agreement, and a small positive bias was observed for total cholesterol, triglycerides, and non-HDL-C, whereas HDL-C showed a slight negative bias, indicating a tendency of the Mission^®^ device to underestimate HDL-C measurements.

In terms of LDL-C estimation, the scatter plots demonstrated a positive linear relationship between the laboratory measurements and the values obtained using the Mission^®^ device, the Martin formula, and the Sampson formula (Figure 2). The Bland–Altman analysis indicated acceptable agreement for all three approaches, although the Mission^®^ device and the Martin equation exhibited narrower dispersion around the mean bias than the Sampson equation, suggesting better agreement with the laboratory reference method.

The agreement statistics are summarized in Table 2, presenting the ICC, CCC, ($R^{2}$), RMSE, MAE, and percent bias values for different lipid profiles measured with the Mission^®^ device and compared with the reference method. Overall, the Mission^®^ device exhibited good agreement with the reference laboratory method for most lipid parameters, although the degree of concordance varied among analytes. Triglycerides showed the strongest performance, with excellent agreement according to both the ICC and CCC, together with the highest coefficient of determination and a low prediction error, indicating highly consistent measurements with the reference method.

For total cholesterol, HDL-C, and non-HDL-C, agreement remained good, with ICC values ranging from 0.817 to 0.846 and CCC values between 0.816 and 0.845. Among these analytes, total cholesterol showed the highest proportion of explained variability, whereas HDL-C exhibited the lowest prediction error, reflecting the greatest measurement accuracy. Although non-HDL-C achieved comparable agreement, it showed slightly higher prediction errors than HDL-C.

For LDL-C, the Mission^®^ device showed good agreement with the reference laboratory method and outperformed both the Martin–Hopkins and Sampson equations in the agreement metrics. Among the calculated methods, the Martin–Hopkins equation consistently demonstrated better concordance and a lower prediction error than the Sampson equation, although neither achieved the level of agreement observed for the Mission^®^ estimates.

Among the evaluated atherogenic ratios, TG/HDL-C and TG/LDL-C demonstrated the highest agreement with the reference method (Figure 3), showing excellent concordance according to both the ICC and CCC, together with the highest coefficients of determination (Table 2), whereas TC/HDL-C and non-HDL-C/HDL-C demonstrated moderate agreement according to the ICC but good concordance based on the CCC. The LDL-C/HDL-C ratio also showed good agreement and the lowest MAE, demonstrating the smallest average absolute error among the evaluated lipid ratios.

### 3.3. Passing–Bablok Regression Analysis

Passing–Bablok regression was used to evaluate the agreement between the Mission device and the laboratory reference method (Table 3). Most lipid profile parameters showed evidence of constant bias, as the 95% CI of the intercept did not include zero. Likewise, proportional bias was observed for most analytes because the 95% CI of the slope did not include one.

For total cholesterol, HDL cholesterol, non-HDL cholesterol, and LDL cholesterol estimated by the Mission^®^ and Martin methods, both constant and proportional bias were present, indicating systematic differences between the Mission measurements and laboratory values. In contrast, triglycerides showed only constant bias, since the slope CI included 1.0, suggesting that, although the measurements consistently shifted, the proportional relationship with the reference method remained preserved.

Among the LDL-C estimation methods, the Mission^®^ device and the Martin equation showed similar patterns, with both constant and proportional biases, whereas the Sampson equation exhibited proportional bias only.

A similar behavior was observed for all evaluated atherogenic ratios. TC/HDL-C, TG/HDL-C, non-HDL-C/HDL-C, and LDL-C/HDL-C all presented evidence of both constant and proportional biases, indicating systematic differences between the Mission^®^ and laboratory-derived ratios despite their strong linear associations.

### 3.4. Accuracy Measures

The diagnostic performance of the Mission^®^ device for classifying lipid abnormalities according to established clinical cut-off values is presented in Table 4. The device demonstrated high sensitivity (94.29–98.33%) and specificity (68.33–91.80%) across all lipid parameters, with area under the receiver operating characteristic curve (AUC) values ranging from 0.916 to 0.989, indicating excellent discrimination between normal and abnormal lipid concentrations.

Triglycerides showed the highest diagnostic performance, with a sensitivity of 96.61%, a specificity of 91.8%, and an AUC of 0.989, reflecting excellent agreement with the laboratory classification and a low frequency of false-positive and false-negative results.

For total cholesterol, the Mission^®^ device achieved a sensitivity of 97.5% and a specificity of 73.75%, with an AUC of 0.961, and HDL-C also demonstrated high sensitivity (96.55%), although specificity was comparatively lower (73.63%), resulting in an AUC of 0.916. Similarly, non-HDL-C showed excellent sensitivity (98.33%) but moderate specificity (68.33%), with an AUC of 0.948.

Mission^®^ LDL-C exhibited excellent diagnostic performance, with a sensitivity of 94.29%, a specificity of 86.00%, and an AUC of 0.970. Comparing these results with alternative LDL-C estimation methods, the Martin equation achieved the same sensitivity as the Sampson equation (95.71%) but higher specificity (82% vs. 72%), whereas Mission yielded the highest AUC among the three approaches. Regarding the atherogenic ratios, TG/HDL-C demonstrated the best overall performance, with a sensitivity of 96.6%, a specificity of 83.61%, and an AUC of 0.977. Non-HDL-C/HDL-C also showed a high sensitivity of 95%, a specificity of 65%, and an AUC of 0.919.

### 3.5. Between-Device Variability

The mixed-effects analysis demonstrated measurable between-device variability across all lipid parameters. The proportion of total variance attributable to between-analyzer differences, as quantified by the device-level intraclass correlation coefficient (ICC_device_), ranged from 0.086 for TG to 0.181 for total cholesterol. The between-device coefficient of variation ranged from 11.51% for total cholesterol to 16.81% for triglycerides, indicating variability in analytical measurements across the evaluated POCT units (Table 5).

## 4. Discussion

The present study shows that the Mission^®^ Cholesterol 3-in-1 device achieved good-to-excellent analytical agreement with the laboratory reference method for most lipid analytes. This finding is clinically relevant because analytical agreement determines whether a point-of-care device can reliably classify patients according to established cardiovascular risk thresholds rather than merely reproduce laboratory measurements. In decentralized settings, diagnostic consistency is often more important than perfect numerical equivalence, since clinical decisions are generally based on predefined cut-off values rather than exact lipid concentrations [47,48].

Our findings show that triglycerides exhibited the strongest analytical performance, achieving excellent agreement (ICC = CCC = 0.972), the lowest root mean square error (RMSE = 19.09 mg/dL), and the smallest positive percent bias (5.19%). These results are consistent with those of previous studies identifying triglycerides as the best-performing analyte of the Mission^®^ system. Kurstjens et al. [19] found that Passing–Bablok regression demonstrated constant bias without proportional bias, a pattern identical to that observed in the present study. Abrahin et al. [20] also identified triglycerides as the lipid parameter with the highest agreement; however, their concordance was substantially lower than that observed in our study (CCC = 0.70 vs. 0.972), and they obtained a higher analytical error (RMSE ≈ 44 mg/dL) and greater absolute bias (31%). Similarly, Quartey et al. [49] reported acceptable analytical precision according to the National Cholesterol Education Program (NCEP) criteria [50] (CV = 3.2–3.23%), although triglyceride bias ranged from 4.3% in normolipidemic samples to 5.3% in dyslipidemic samples, exceeding the NCEP allowable bias in the latter group. By contrast, the Mission^®^ system in our study demonstrated the smallest positive percent bias (5.19%), remaining within the NCEP analytical performance limits. In addition, Bland–Altman analyses, together with bias estimates and prediction error metrics, have consistently indicated a slight overestimation of triglyceride concentrations in previous validation studies [19,20,49]. Our findings corroborate this observation, as triglycerides also exhibited a small positive systematic bias. Although direct comparisons among studies should be interpreted cautiously because of differences in study design, study populations, and analytical methodology, the consistent observation that triglycerides are the best-performing analyte, together with the superior agreement, lower analytical error, and smaller bias observed in the present study, further supports the robustness and reliability of Mission^®^ triglyceride measurements across diverse clinical settings.

Total cholesterol, HDL-C, non-HDL-C, and LDL-C also demonstrated good analytical agreement, and these findings are partially consistent with those of previous studies. Kurstjens et al. [19] reported a similar coefficient of determination for total cholesterol ($R^{2}$ = 0.921). In the present study, the coefficients of determination were 0.848 for total cholesterol and 0.797 for HDL-C. Likewise, Abrahin et al. [20] reported concordance correlation coefficients of 0.89 for total cholesterol, 0.81 for HDL-C, and 0.85 for LDL-C, values that are comparable to those observed in our study. Together, these findings suggest that the analytical performance of the Mission^®^ system for total cholesterol, HDL-C, and LDL-C is reproducible in different populations and validation studies. Although agreement metrics vary slightly across investigations, likely due to differences in sample characteristics and study design, the consistent demonstration of good analytical agreement supports use of the device as a reliable point-of-care tool for lipid detection. Furthermore, the present study extends previous evidence by demonstrating this performance in a larger, more heterogeneous population using a comprehensive analytical validation framework.

Analytical bias also compared favorably with that in previous reports. Kurstjens et al. [19] reported absolute biases of +19% for total cholesterol, −8% for HDL-C, and +20% for LDL-C, whereas the corresponding values in our study were +8.33%, −6.18%, and +6.05%, respectively. Similarly, Perez Quartey et al. [49] reported a total cholesterol bias of 11.1% in normolipidemic samples, exceeding the NCEP allowable limits [50], although HDL-C bias remained low (2.2%). In dyslipidemic samples, total cholesterol and HDL-C biases were 3.5% and 3.0%, respectively. Abrahin et al. [20] also reported similar RMSE values for total cholesterol (24 vs. 26.20 mg/dL in the present study), whereas our study demonstrated lower RMSE values for HDL-C (8.55 vs. 10 mg/dL) and LDL-C (20.74 vs. 25 mg/dL), suggesting improved analytical precision. In addition, Bland–Altman analyses, bias estimates, and prediction error metrics consistently demonstrated a slight overestimation of total cholesterol and LDL-C, together with a slight underestimation of HDL-C, in previous validation studies [19,20,49]. Our findings are consistent with this pattern, confirming similar systematic biases for these analytes. Differences were also observed in the Passing–Bablok regression analysis. Kurstjens et al. [19] reported proportional bias without constant bias for total cholesterol and LDL-C, whereas HDL-C exhibited constant bias without proportional bias. In contrast, our study demonstrated both constant and proportional biases for all three analytes. Despite these systematic differences, HDL-C remained within the analytical performance limits established by the NCEP [50]. This finding is clinically relevant because elevated triglycerides and reduced HDL-C constitute two diagnostic components of metabolic syndrome. Therefore, in addition to conventional assessment of dyslipidemia, the Mission^®^ system may represent a useful tool for cardiometabolic screening. However, because metabolic syndrome was not evaluated as a clinical outcome in the present study, this potential application should be confirmed in specifically designed prospective studies.

The Mission^®^ Cholesterol 3-in-1 system demonstrated good-to-excellent diagnostic performance across the evaluated lipid parameters, with sensitivities ranging from 94.3% to 98.3%, specificities from 68.3% to 91.8%, and areas under the ROC curve (AUCs) between 0.916 and 0.989. These findings indicate that, despite the small systematic biases identified by Passing–Bablok regression, the device maintains a high ability to correctly classify individuals according to clinically relevant lipid thresholds. Consequently, the observed analytical differences between the Mission^®^ system and the laboratory reference method are unlikely to have a meaningful impact on its performance as a screening tool for dyslipidemia and cardiovascular risk assessment. Our results are consistent with those of previous studies evaluating the clinical performance of portable lipid analyzers. For example, Kurstjens et al. [19] reported excellent sensitivity (96%) and moderate specificity (67%) for total cholesterol. In comparison, the present study demonstrated slightly higher sensitivity (97.5%) and specificity (73.8%), further supporting the use of the Mission^®^ system as a reliable alternative for cholesterol screening in appropriate clinical settings. The comparative data presented in Table 6 further contextualize the diagnostic performance of the Mission^®^ Cholesterol 3-in-1 system relative to that of other POCT devices. For total cholesterol, the Mission^®^ system showed a higher sensitivity and specificity than those reported by Kurstjens et al. [19] (97.5% vs. 96% and 73.8% vs. 67%, respectively), while its performance was also characterized by substantially higher sensitivity than that reported for the CardioChek® PA (57.1%) and Cholestech LDX (79%) [51,52]. For triglycerides, the sensitivity and specificity observed with the Mission^®^ system (96.6% and 91.8%) were higher than those reported for the CardioChek® PA (76.0% and 100%), Accutrend Plus (90.5% and 96.9%), and Cholestech LDX (88% and 93%) [51,52,53]. The Mission^®^ system also demonstrated high sensitivity for HDL-C and LDL-C (96.6% and 94.3%, respectively), exceeding the corresponding sensitivities reported for the CardioChek® PA and Cholestech LDX. However, these comparisons should be interpreted cautiously because the studies differed in the sample size, specimen type, analytical procedures, and clinical thresholds used for classification. In particular, the present study used venous whole blood, whereas most previous investigations evaluated capillary blood, which may contribute to the differences observed in diagnostic performance. Overall, the findings support the potential clinical utility of the Mission^®^ system for lipid screening while emphasizing that diagnostic performance estimates are context-dependent and should be interpreted alongside analytical agreement and clinical applicability.

An unexpected finding was that the LDL-C values generated directly by the Mission^®^ system demonstrated better agreement than the LDL-C values recalculated using the Martin–Hopkins or Sampson equations from the same measurements. This suggests that the manufacturer’s patented calibration algorithm can partially compensate for the analytical variability introduced by portable measurements; additionally, it can even achieve better agreement than the manual Friedewald formula: the authors of [49] reported an R^2^ value of 0.99, and we calculated a value of 0.837.

Among the atherogenic ratios evaluated, TG/HDL-C and TG/LDL-C showed the best analytical performance, with excellent agreement with the reference method and low predictive errors. The Passing–Bablok regression analysis showed that the TG/HDL-C ratio showed constant and proportional biases, while the TG/LDL-C ratio did not show either of these types of biases, suggesting excellent analytical stability of the latter. Furthermore, TG/HDL-C presented high sensitivity (96.61%) and good specificity (83.61%), while TG/LDL-C showed moderate sensitivity (80.95%) but the highest specificity (96.12%) among the ratios evaluated. This is probably because the analytes that compose them (triglycerides, HDL-C, and LDL-C) individually showed good-to-excellent agreement with the reference method, allowing the derived ratios to maintain adequate analytical and diagnostic performance. These findings have clinical relevance, since atherogenic ratios integrate information from multiple lipid fractions and have been proposed as markers of insulin resistance, metabolic syndrome, and cardiovascular risk [12,36,54]. Consequently, demonstrating that these ratios can be reliably estimated using a portable point-of-care system expands the potential applications of these devices beyond the evaluation of isolated lipid parameters, favoring their use as cardiometabolic screening tools in settings where laboratory access is limited. However, prospective studies are necessary to confirm whether the ratios obtained using POCT devices retain their prognostic capacity for cardiometabolic events and metabolic syndrome.

The observed between-device variability indicates that analytical performance was not fully consistent across the evaluated Mission^®^ units. The between-device coefficient of variation was highest for triglycerides (16.81%) and LDL-C (15.10%), whereas total cholesterol showed the lowest variability (11.51%). However, the proportion of total variance attributable to between-device differences was relatively low across all lipid parameters, with ICC_device_ values ranging from 0.086 for triglycerides to 0.181 for total cholesterol. These findings indicate that, although measurable differences existed among the evaluated POCT units, most of the observed variability was attributable to within-device or residual variation rather than differences between devices.

The present study has several methodological strengths. The evaluation of eleven independent Mission^®^ analyzers reduces the likelihood that the observed results reflect the performance of a single instrument and allowed the assessment of between-device variability across the evaluated units. In addition, the use of complementary analytical methods, including agreement coefficients, error metrics, bias assessment, regression analysis, diagnostic accuracy measures, and between-device variability analysis, allowed device performance to be assessed from multiple perspectives. Although each of these approaches has been reported previously, their integration within a single validation study provides a broader characterization of the analytical performance of the Mission^®^ Cholesterol system.

This study presents certain limitations. First, the sample size, while adequate for an agreement study, limited the possibility of conducting detailed subgroup analyses. Second, participants were recruited from a single center, and measurements were performed under controlled environmental conditions, which may limit the generalizability of the findings to other populations and routine point-of-care settings. Third, within-device repeatability and measurement stability over time were not assessed because of the cross-sectional design. Fourth, although the device demonstrated good agreement and high diagnostic capability, Passing–Bablok analysis revealed systematic bias for most analytes; therefore, its measurements should not be considered fully interchangeable with those of the reference method when maximum analytical accuracy is required. Finally, the Mission^®^ system was evaluated using venous whole blood rather than capillary fingerstick blood. Therefore, the present findings primarily describe analytical agreement under venous whole-blood sampling conditions and cannot be directly extrapolated to capillary sampling, which represents an important operational setting for POCT screening. Future studies should specifically evaluate the performance of the system using capillary fingerstick samples under routine point-of-care conditions.

## 5. Conclusions

In conclusion, the Mission^®^ Cholesterol 3-in-1 system demonstrated good analytical agreement and favorable diagnostic performance compared with the laboratory reference method. Triglycerides, together with the TG/HDL-C and TG/LDL-C atherogenic ratios, exhibited the strongest analytical performance, whereas total cholesterol, HDL-C, non-HDL-C, and LDL-C showed acceptable agreement. Although the presence of systematic bias precludes complete analytical interchangeability with standardized laboratory methods, the overall analytical and diagnostic performance of the Mission^®^ system supports its use as a reliable point-of-care tool for lipid profile screening, cardiovascular risk assessment, epidemiological studies, and clinical follow-up, particularly in healthcare settings where rapid testing and limited access to conventional laboratory services make portable lipid analyzers especially valuable.

## Figures and Tables

**Figure 1:**
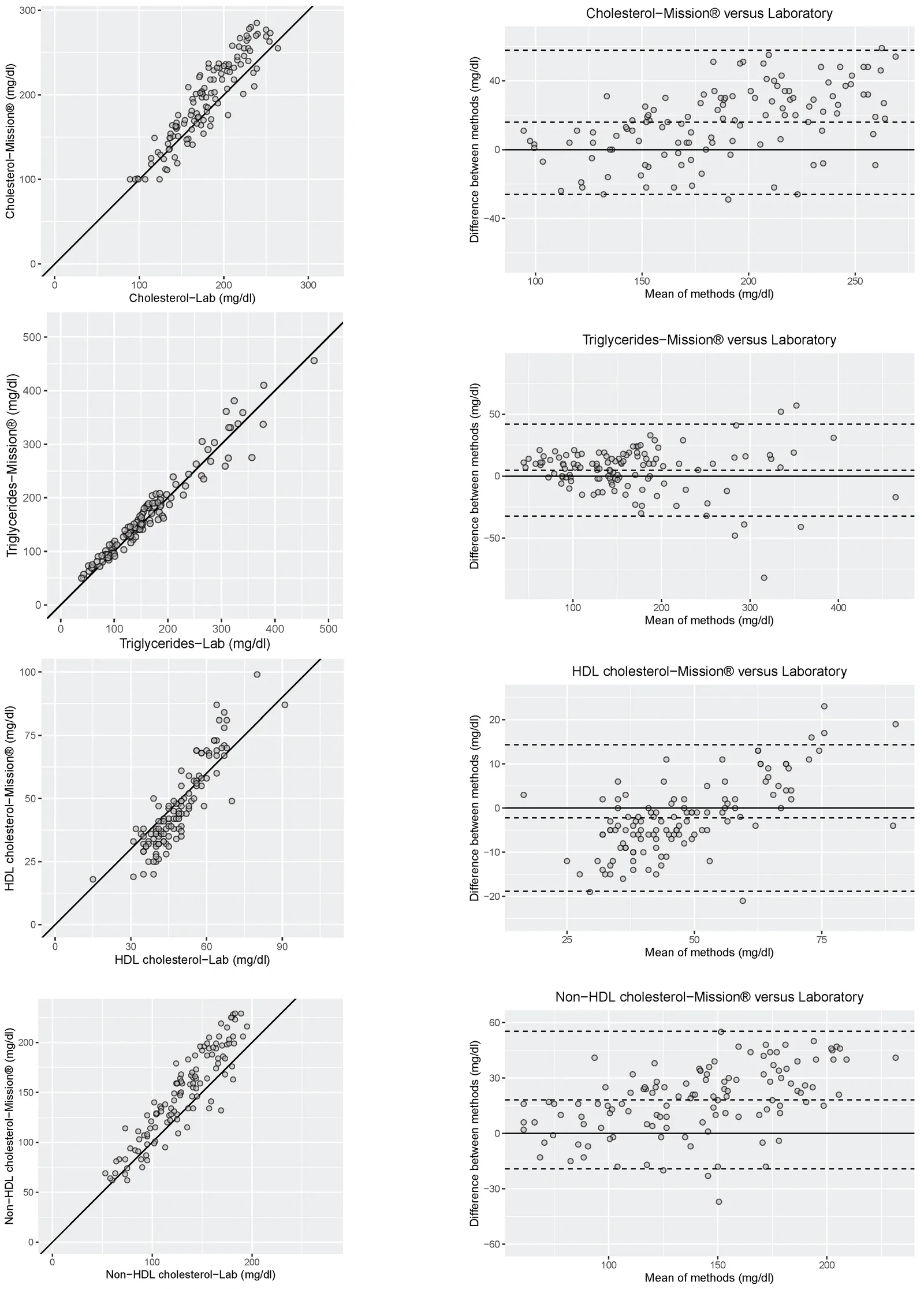
Scatter plots and Bland–Altman plots of lipid profile measurements using the Mission vs. laboratory methods. In the Bland–Altman plots, the solid line represents the mean difference (bias), and the dotted lines represent the 95% limits of agreement.

**Figure 2:**
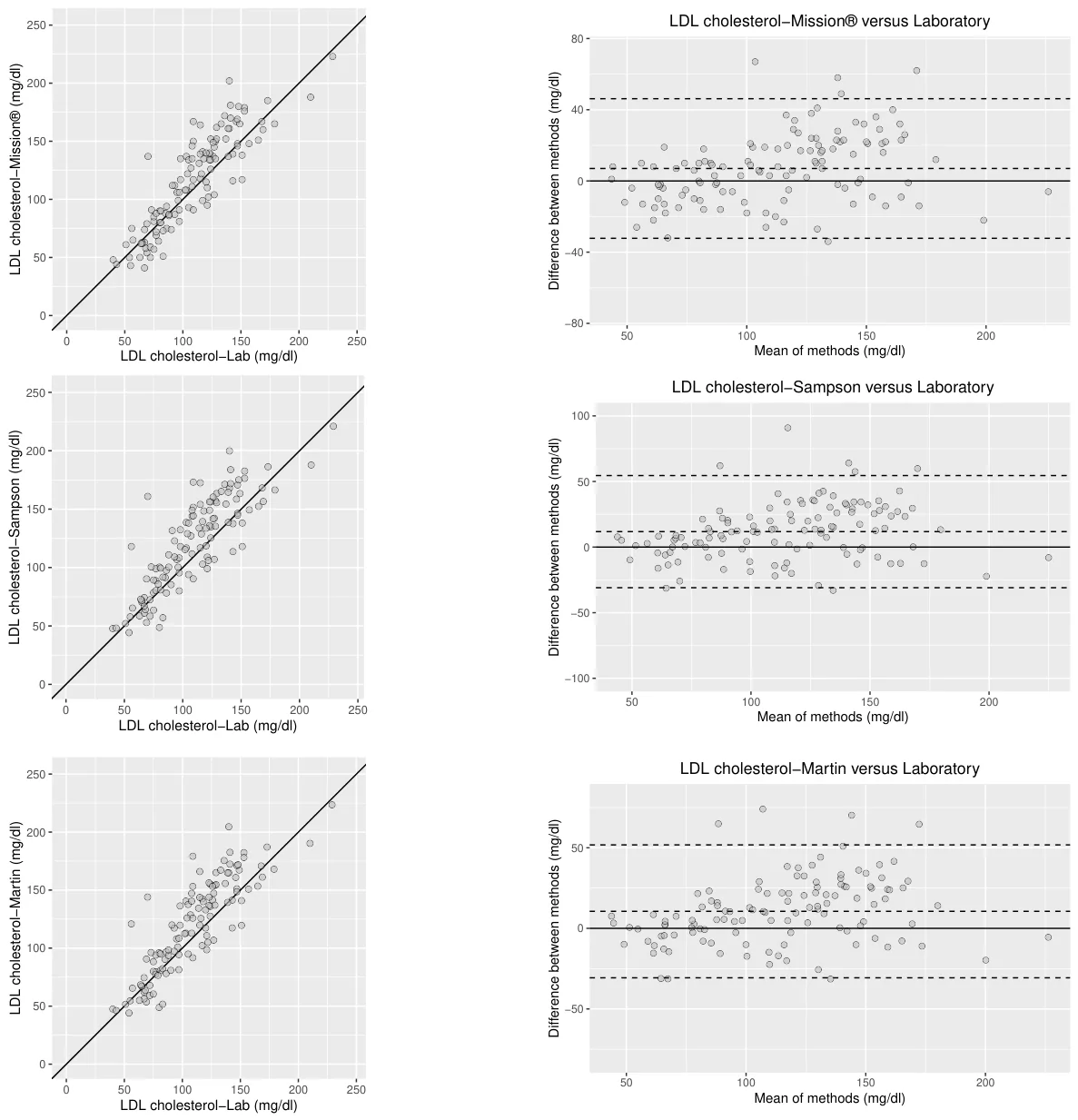
Scatter plots and Bland–Altman plots of LDL-C measurements obtained using the formulas of Friedewald, Sampson, and Martin. In the Bland–Altman plots, the solid line represents the mean difference (bias), and the dotted lines represent the 95% limits of agreement.

**Figure 3:**
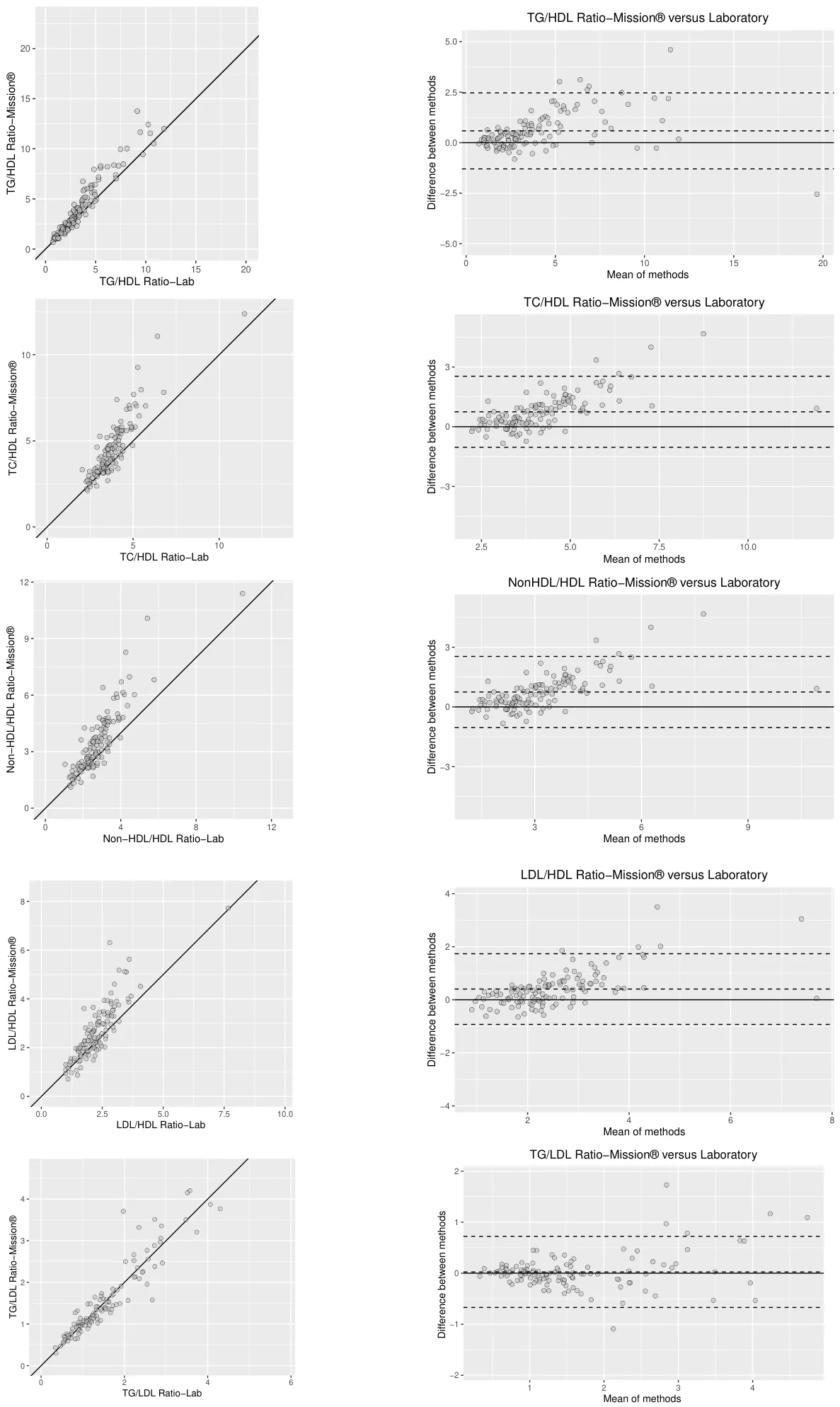
Scatter plots and Bland–Altman plots of lipid ratios using the Mission vs. laboratory methods. In the Bland–Altman plots, the solid line represents the mean difference (bias), and the dotted lines represent the 95% limits of agreement.

**Table 1:** Biochemical and clinical characteristics of patients.

Characteristics	*n* = 120
Age (years), mean (SD)	49.01 (19.39)
Sex (female), f (%)	77 (64.2%)
Hypertriglyceridemia (≥150 mg/dL), f (%)	59 (49.2%)
Hypercholesterolemia (≥200 mg/dL), f (%)	40 (33.3%)
HDL-C (<40 mg/dL), f (%)	29 (24.2%)
Non-HDL-C (≥130 mg/dL), f (%)	60 (50.0%)
LDL-C (≥100 mg/dL), f (%)	70 (58.3%)
Type 2 diabetes, f (%)	42 (35.0%)
Arterial hypertension, f (%)	7 (5.8%)
Lipid profile	
Total cholesterol (mg/dL), mean (SD)	180.02 (40.27)
Triglycerides (mg/dL), median (Q1, Q3)	149.0 (101.0, 187.0)
HDL-C (mg/dL), mean (SD)	48.89 (11.26)
Non-HDL-C (mg/dL), mean (SD)	131.1 (36.1)
LDL-C (mg/dL), mean (SD)	108.8 (34.6)
Atherogenic ratios	
TC/HDL-C, median (Q1, Q3)	3.67 (3.22, 4.24)
TG/HDL-C, median (Q1, Q3)	2.96 (2.01, 4.54)
Non-HDL-C/HDL-C, median (Q1, Q3)	2.67 (2.21, 3.24)
LDL-C/HDL-C, median (Q1, Q3)	2.19 (1.78, 2.71)
TG/LDL-C, median (Q1, Q3)	1.34 (0.928, 2.05)

SD: standard deviation; Q1: first quartile; Q3: third quartile; *n* (%): absolute and relative frequency; HDL-C: high-density lipoprotein cholesterol; LDL-C: low-density lipoprotein cholesterol; TC: total cholesterol; TG: triglycerides.

**Table 2:** Concordance between lipid profile and atherogenic ratios measured by the Mission^®^ device and standard laboratory tests.

Lipid Profile	ICC	CCC	R^2^	RMSE	MAE	Percent Bias (%)
Total cholesterol (Mission^®^)	0.846 (0.807, 0.879)	0.845 (0.803, 0.879)	0.848 (0.784, 0.886)	26.20 (23.59, 29.23)	21.85 (19.39, 24.55)	8.33 (6.19, 10.07)
Triglycerides (Mission^®^)	0.972 (0.960, 0.981)	0.972 (0.959, 0.981)	0.949 (0.918, 0.965)	19.09 (16.46, 24.53)	14.49 (12.51, 17.02)	5.19 (3.18, 7.22)
HDL-C (Mission^®^)	0.820 (0.775, 0.867)	0.819 (0.774, 0.866)	0.797 (0.707, 0.846)	8.55 (7.59, 9.61)	6.88 (6.01, 7.84)	−6.180 (−9.21, −3.27)
Non-HDL-C (Mission^®^)	0.817 (0.775, 0.855)	0.816 (0.774, 0.854)	0.844 (0.774, 0.889)	25.91 (23.63, 28.40)	22.05 (19.73, 24.70)	13.70 (11.10, 16.10)
LDL-C (Mission^®^)	0.854 (0.796, 0.896)	0.852 (0.794, 0.895)	0.776 (0.683, 0.832)	20.74 (18.25, 24.61)	16.36 (14.26, 18.89)	6.05 (2.85, 9.65)
LDL-C (Sampson)	0.797 (0.711, 0.858)	0.798 (0.709, 0.857)	0.712 (0.582, 0.785)	24.33 (21.33, 29.28)	18.93 (16.41, 21.90)	11.78 (6.52, 14.16)
LDL-C (Martin)	0.821 (0.744, 0.876)	0.821 (0.743, 0.875)	0.745 (0.634, 0.813)	23.08 (20.01, 27.09)	17.75 (15.13, 20.76)	10.04 (6.52, 14.16)
Atherogenic ratios						
TC/HDL-C (Mission^®^)	0.709 (0.607, 0.863)	0.707 (0.605, 0.862)	0.768 (0.684, 0.837)	1.16 (0.983, 1.483)	0.845 (0.722, 1.029)	18.45 (15.02, 22.18)
TG/HDL-C (Mission^®^)	0.932 (0.894, 0.963)	0.931 (0.893, 0.963)	0.911 (0.871, 0.932)	1.10 (0.929, 1.41)	0.748 (0.625, 0.930)	15.26 (11.75, 19.14)
Non-HDL-C/HDL-C (Mission^®^)	0.709 (0.606, 0.863)	0.707 (0.605, 0.862)	0.767 (0.684, 0.837)	1.16 (0.983, 1.483)	0.845 (0.722, 1.02)	25.29 (20.61, 30.44)
LDL-C/HDL-C (Mission^®^)	0.765 (0.655, 0.867)	0.763 (0.653, 0.866)	0.764 (0.627, 0.843)	0.776 (0.631, 1.01)	0.533 (0.440, 0.655)	15.82 (11.53, 20.68)
TG/LDL-C (Mission^®^)	0.931 (0.875, 0.953)	0.929 (0.875, 0.953)	0.875 (0.781, 0.914)	0.347 (0.279, 0.473)	0.219 (0.178, 0.278)	2.07 (−1.13, 5.81)

ICC: intraclass correlation coefficient; CCC: concordance correlation coefficient; R^2^: coefficient of determination; RMSE: root mean squared error; MAE: mean absolute error. Values in parentheses represent 95% confidence intervals; HDL-C: high-density lipoprotein cholesterol; LDL-C: low-density lipoprotein cholesterol; TC: total cholesterol; TG: triglyceride.

**Table 3:** Passing–Bablok regression analysis between Mission^®^ measurements and laboratory reference measurements for lipid profile parameters and atherogenic ratios.

Parameter	Intercept (95% CI)	Slope (95% CI)	Constant Bias	Proportional Bias
**Lipid profile**				
Total cholesterol (Mission^®^)	−33.60 (−50.38, −18.63)	1.301 (1.209, 1.387)	Yes	Yes
Triglycerides (Mission^®^)	9.00 (3.32, 13.90)	1.000 (0.951, 1.036)	Yes	No ^a^
HDL-C (Mission^®^)	−29.83 (−36.75, −23.08)	1.556 (1.417, 1.700)	Yes	Yes
Non-HDL-C (Mission^®^)	−14.41 (−26.09, −4.31)	1.273 (1.188, 1.364)	Yes	Yes
LDL-C (Mission^®^)	−20.39 (−33.47, −8.58)	1.263 (1.151, 1.389)	Yes	Yes
LDL-C (Sampson)	8.91 (−0.81, 17.83)	0.809 (0.732, 0.905)	No	Yes
LDL-C (Martin)	−16.98 (−29.33, −5.38)	1.267 (1.150, 1.385)	Yes	Yes
**Atherogenic ratios**				
TC/HDL-C (Mission^®^)	−2.00 (−2.70, −1.33)	1.735 (1.546, 1.932)	Yes	Yes
TG/HDL-C (Mission^®^)	−0.269 (−0.547, −0.075)	1.264 (1.186, 1.353)	Yes	Yes
Non-HDL-C/HDL-C (Mission^®^)	−1.263 (−1.764, −0.780)	1.735 (1.546, 1.932)	Yes	Yes
LDL-C/HDL-C (Mission^®^)	−0.875 (−1.211, −0.494)	1.567 (1.390, 1.749)	Yes	Yes
TG/LDL-C (Mission^®^)	0.004 (−0.096, 0.086)	0.992 (0.924, 1.068)	No	No

CI: confidence interval. Constant bias was considered present when the 95% confidence interval (CI) of the intercept did not include zero. Proportional bias was considered present when the 95% CI of the slope did not include one. ^a^ The 95% CI of the slope included 1.00; therefore, proportional bias was not considered statistically significant; HDL-C: high-density lipoprotein cholesterol; LDL-C: low-density lipoprotein cholesterol; TC: total cholesterol; TG: triglyceride.

**Table 4:** Diagnostic accuracy of lipid profile and atherogenic ratios dichotomized by clinical cutoff values.

Lipid Profile	TP	FN	TN	FP	Se (%)	Sp (%)	AUC
Total cholesterol (Mission^®^)	39	1	59	21	97.50 (86.84, 99.94)	73.75 (62.71, 82.96)	0.961 (0.930, 0.992)
Triglycerides (Mission^®^)	57	2	56	5	96.61 (88.29, 99.59)	91.80 (81.90, 97.28)	0.989 (0.976, 1.00)
HDL-C (Mission^®^)	28	1	67	24	96.55 (82.24, 99.91)	73.63 (63.35, 82.31)	0.916 (0.862, 0.970)
Non-HDL-C (Mission^®^)	59	1	41	19	98.33 (91.06, 99.96)	68.33 (55.04, 79.74)	0.948 (0.914, 0.982)
LDL-C (Mission^®^)	66	4	43	7	94.29 (86.01, 98.42)	86.00 (73.26, 94.18)	0.970 (0.945, 0.996)
LDL-C (Sampson)	67	3	36	4	95.71 (87.98, 99.11)	72.00 (57.51, 83.77)	0.951 (0.913, 0.988)
LDL-C (Martin)	67	3	41	9	95.71 (87.98, 99.11)	82.00 (68.56, 91.42)	0.961 (0.930, 0.991)
**Atherogenic ratios**							
TG/HDL-C (Mission^®^)	57	2	51	10	96.61 (88.29, 99.59)	83.61 (71.91, 91.85)	0.977 (0.956, 0.999)
TC/HDL-C (Mission^®^)	42	1	52	25	97.67 (87.71, 99.94)	67.53 (55.90, 77.77)	0.949 (0.911, 0.988)
Non-HDL-C/HDL-C (Mission^®^)	57	3	39	21	95.00 (86.08, 98.96)	65.00 (51.60, 76.87)	0.919 (0.874, 0.965)
LDL-C/HDL-C (Mission^®^)	56	6	41	17	90.32 (80.12, 96.37)	70.69 (57.27, 81.91)	0.895 (0.841, 0.950)
TG/LDL-C (Mission^®^)	17	4	99	4	80.95 (58.09, 94.55)	96.12 (90.35, 98.93)	0.971 (0.943, 0.999)

Values in parentheses represent 95% confidence intervals. TP: true positive; FN: false negative; TN: true negative; FP: false positive; Se: sensitivity; Sp: specificity; AUC: area under the curve; HDL-C: high-density lipoprotein cholesterol; LDL-C: low-density lipoprotein cholesterol; TC: total cholesterol; TG: triglyceride.

**Table 5:** Between -device variability for lipid profile measurements obtained with the Mission^®^ Cholesterol 3-in-1 system.

Lipid Profile	Var_between−device_	Var_within−device_	ICC_device_	CV_between−device_ (%)
Total cholesterol (Mission^®^)	72.910	330.433	0.181	11.51
Triglycerides (Mission^®^)	28.917	306.359	0.086	16.81
HDL-C (Mission^®^)	5.208	51.651	0.092	11.90
LDL-C (Mission^®^)	46.860	341.461	0.121	15.10

Var_between−device_: variance component attributable to differences between analyzers; Var_within−device_: residual variance component; ICC_device_: device-level intraclass correlation coefficient; CV_between−device_: coefficient of variation between devices.

**Table 6:** Diagnostic performance of point-of-care testing devices for lipid profile measurements in previous and present studies.

Device/Study	*n*	POCT Specimen	TC Se/Sp (%)	TG Se/Sp (%)	HDL-C Se/Sp (%)	LDL-C Se/Sp (%)
Mission^®^ 3-in-1,						
Present study	120	Venous whole blood	97.5/73.75	96.61/91.80	96.55/73.63	94.29/86.00
CardioChek^®^ PA [51]	203	Capillary blood (fingerstick)	57.1/94.3	76.0/100	96.2/83.2	81.0/100
Mission^®^ 3-in-1 [19]	62	Capillary blood	96/67	NA	NA	NA
Accutrend Plus [19]	62	Capillary blood	92/89	NA	NA	NA
Accutrend Plus [53]	53	Capillary blood	84.4/95.2	90.5/96.9	NA	NA
Cholestech LDX [52]	250	Capillary blood	79/95	88/93	93/78	93/82

Se: sensitivity; Sp: specificity; TC: total cholesterol; TG: triglyceride; HDL-C: high-density lipoprotein cholesterol; LDL-C: low-density lipoprotein cholesterol; POCT: point-of-care testing; NA: not available. Values are presented as sensitivity/specificity (%).

## Data Availability

The data presented in this study are available in the article.

## Abbreviations

The following abbreviations are used in this manuscript:

AHA	American Heart Association
AUC	Area Under the Curve
CCC	Concordance Correlation Coefficient
CDMX	Mexico City
CVD	Cardiovascular Disease
FN	False Negative
FP	False Positive
HDL-C	High-Density Lipoprotein Cholesterol
ICC	Intraclass Correlation Coefficient
IDL	Intermediate Density Lipoprotein
LDL-C	Low-Density Lipoprotein Cholesterol
MAE	Mean Absolute Error
non-HDL-C	Non-High-Density Lipoprotein Cholesterol
POCT	Point-of-Care Testing Device
Q1, Q3	First and Third Quartiles
R^2^	Coefficient of Determination
RMSE	Root Mean Square Error
SD	Standard Deviation
TC	Total Cholesterol
TG	Triglyceride
VLDL	Very Low-Density Lipoprotein
WHO	World Health Organization
